# Supplementary material for: Low Magnesium in Conjunction with High Homocysteine and Less Sleep Accelerates Telomere Attrition in Healthy Elderly Australian
Source: Int J Mol Sci. 2023 Jan 4;24(2):982. doi: 10.3390/ijms24020982 (PMC9866301; doi:10.3390/ijms24020982)
Supplement: Supplementary file 1 [file ijms-24-00982-s001.zip › ijms-2070951-supplementary.pdf]

Supplementary Figure S1: Bivariate analysis of Homocysteine and Magnesium.

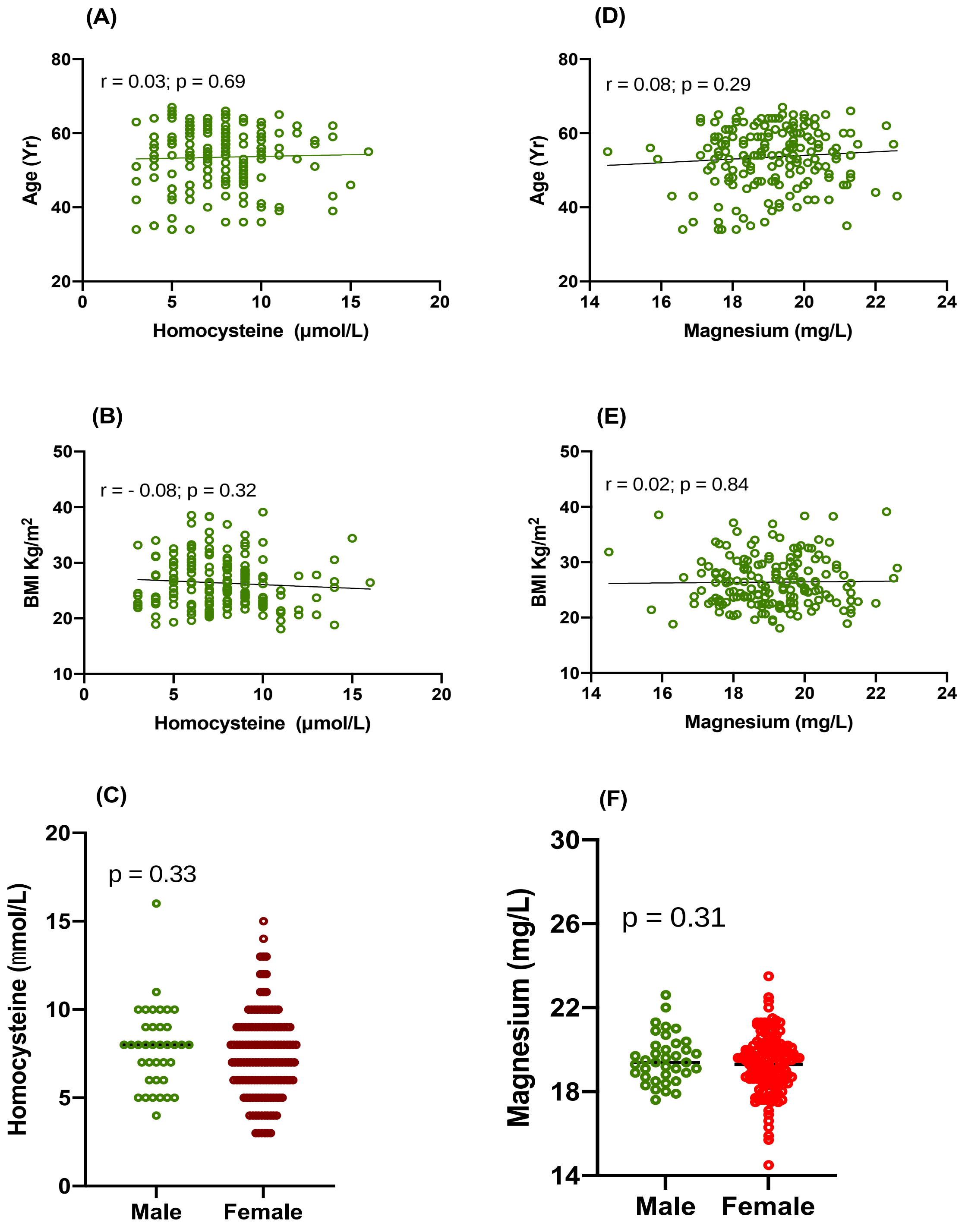

(**A**) The correlation of homocysteine with Age; (**B**) correlation of homocysteine with BMI; (**C**) homocysteine concentration in males and females; (**D**) correlation of magnesium with age; (**E**) correlation of magnesium with BNI; and (**F**) magnesium concentration in males and females.
